# 以血管外容量负荷增大为首诊表现的POEMS综合征临床特征：单中心回顾性研究

**DOI:** 10.3760/cma.j.cn121090-20231117-00264

**Published:** 2024-07

**Authors:** 婷 张, 珏 张, 旭星 沈, 媛媛 金, 闰 张, 建勇 李, 丽娟 陈

**Affiliations:** 南京医科大学第一附属医院，江苏省人民医院血液科，南京 210029 Department of Hematology, the First Affiliated Hospital of Nanjing Medical University, Jiangsu Province Hospital, Nanjing 210029, China

## Abstract

POEMS综合征是一种罕见的浆细胞恶液质，临床表现包括多发性神经病、单克隆免疫球蛋白病、血管外容量负荷增大、内分泌病、脏器肿大和皮肤改变等。由于患者多系统受累，就诊时复杂不典型的症状使得早期诊断面临挑战。该病首发症状以周围神经病变——肢体麻木最常见，而关于血管外容量负荷增大的病例报道少见。研究收集并分析两组不同首发症状（血管外容量负荷增大、肢体麻木）患者的临床资料，总结其临床特点和治疗反应。

## Abstract (en)

POEMS syndrome is a rare plasma cell dysplasia. Its clinical manifestations include polyneuropathy, monoclonal protein, increased extravascular volume load, endocrinopathy, organomegaly and skin changes. The complex and atypical symptoms at presentation make early diagnosis challenging due to multiple system involvement. Peripheral neuropathy, limb numbness, is the most common initial symptom of this disease. However, case reports of increased extravascular volume load are rare. This article collected and analyzed the clinical data of two groups of patients with different initial symptoms（increased extravascular volume load and limb numbness）. The clinical characteristics and treatment responses were summarized.

POEMS综合征是一种罕见的多系统病变疾病。尽管首发症状各异，但通常以某个单一症状展现，且在早期，多系统的损伤可能并不明显。这种低发病率和隐蔽的起病方式使得患者经常在不同专科间频繁就诊。当前临床医师普遍缺乏对这种疾病的认知，误诊的情况相当普遍[1]。特别是那些以腹胀、下肢水肿或胸闷等为首发症状的患者，症状特异性差，需要与其他常见病鉴别。这类患者发病早期常被忽视，确诊延迟长达12～16个月，而此时多数患者的病情可能已经恶化到严重残疾。鉴于这一情况的严峻性，我国医学界已开始加强对其的关注。

目前关于POEMS综合征的研究主要集中在病例回顾性分析和个案报道，首发症状以周围神经病变肢体麻木最常见，对于该病的血管外容量负荷异常方面的报道相对较少[2]。为了进一步了解和探讨这一现象，本研究通过比较血液科收治的17例以血管外容量负荷增大为首发症状的患者与26例以肢体麻木为首发症状的患者，更为详细地描述前者的临床特点，尝试揭示其特征规律，从而加强对以血管外容量负荷增加为首发症状的POEMS综合征的认识，并强调早期诊断与治疗的重要性。

## 病例与方法

1. 病例：对江苏省南京医科大学第一附属医院血液科在2010年1月至2023年9月确诊的、以血管外容量负荷增大或肢体麻木为首发症状的POEMS综合征患者的临床资料，如年龄、性别、病史、体征、实验室检查、治疗等进行回顾性分析。经严格筛选共纳入43例患者，男24例，女19例。本研究经我院伦理委员会批准（批件号：2020-SR-589），并获得患者的知情同意。

2. 方法：本文采用Dispenzieri于2019年提出的诊断标准[3]，典型的POEMS综合征患者需满足多发性神经病变和单克隆浆细胞增殖性异常的强制性标准，以及至少1条主要标准和1条次要标准；未完全符合上述强制性标准的POEMS综合征归纳为变异型[4]–[6]。主要标准包括硬化性骨病、Castleman病和血管内皮生长因子（VEGF）水平升高；次要标准包括脏器（肝、脾、淋巴结）的肿大，水肿（表现为肢体、胸腔或腹腔积液），内分泌代谢异常（可能涉及多个腺体），皮肤的各种改变，视乳头水肿或血小板增多症。参照个体化预后模型[7]：年龄>50岁（1分）、胸腔积液（1分）、肺动脉高压（1分）、肾小球滤过率（eGFR）<30 ml·min^−1^·1.73 m^−2^（2分），将患者分为低危（0分）、中危（1分）、高危（2～5分）。

3. 随访：采用查阅临床资料或电话随访方式，随访时间截至2023年9月30日。患者自确诊开始治疗为随访起点，以死亡或随访结束为随访终点。

4. 统计学处理：应用SPSS 27.0.1软件进行统计学分析。计量资料采用*M*（范围）表示，计数资料采用频数及百分率表示。

## 结果

1. 一般资料：以血管外容量负荷增大为首发症状的患者共17例，男9例，女8例，中位年龄为57（21～75）岁，男性患者中位年龄64（21～75）岁，女性患者中位年龄52.5（35～65）岁；以肢体麻木为首发症状的患者共26例，男15例，女11例，中位年龄为53.5（34～72）岁，男性患者中位年龄55（34～72）岁，女性患者中位年龄51（49～71）岁。

血管外容量负荷增大组患者的首诊科室主要集中于血液内科（35.29％）、肾脏内科（29.41％）、消化内科（11.76％），少数患者于风湿科、神经内科、内分泌科、全科就诊。这些患者自发病至确诊的中位时间长达12（1～60）个月，其中≥12个月确诊的有9例（52.94％）。而肢体麻木组患者首诊则集中于血液内科（42.31％）、神经内科（34.62％）、风湿科（11.54％），内分泌科、肾脏内科占少数。该组患者确诊的中位时间是8（1～96）个月，其中≥12个月确诊的有9例（34.62％）。

2. 临床表现：血管外容量负荷增大组的首诊主诉包括腹胀（52.94％）、下肢水肿（52.94％）、颜面水肿（29.41％）、胸闷（5.88％），其中主诉伴下肢麻木占23.53％。回顾性分析患者的临床特征，14例（82.35％）有肢体麻木、疼痛、乏力等多发性神经病变症状，10例（58.82％）肌电图显示上下肢周围神经损害，4例（23.53％）患有硬化性骨病。7例（41.18％）患者已检测VEGF，结果均超出正常范围，中位数为739.7（374.9～1 235.0）pg/ml。研究纳入的17例患者全部出现了不同程度的脏器肿大（肝肿大23.53％、脾肿大76.47％、淋巴结肿大82.35％），其中8例（47.06％）完成淋巴结穿刺活检，3例（17.65％）确诊Castleman病。11例（64.71％）出现肢端凹陷性水肿，12例（70.59％）腹水阳性，10例（58.82％）胸腔积液阳性，12例（70.59％）心包积液阳性，多为轻、中度积液，2例（11.76％）视乳头水肿，12例（70.59％）存在皮肤色素沉着，3例（17.65％）皮肤干燥粗糙，3例（17.65％）出现皮肤血管瘤，5例（29.41％）性功能异常，3例（17.65％）糖耐量异常。17例（100％）全部出现内分泌改变，累及内分泌轴（甲状腺、甲状旁腺、肾上腺皮质、胰腺、垂体、性腺）数量≤2个的患者有5例（29.41％），数量>2个的患者有12例（70.59％）。

血清免疫电泳M蛋白阳性率64.71％（11/17），均为λ型（IgA型7例，IgG型3例，未检测出重链型1例）。17例行骨髓涂片，12例（70.59％）同时行穿刺活检，骨髓涂片浆细胞占比<2％占100％，骨髓活检中2例（16.67％）浆细胞增多。按个体化预后模型分类，低危1例（5.88％），中危11例（64.71％），高危5例（29.41％）。

肢体麻木组24例（92.31％）肌电图显示周围神经受损，22例（84.62％）有血管外容量负荷增加。10例（38.46％）患有硬化性骨病，15例患者（57.69％）已检测VEGF，结果均升高，中位数为582.0（340.2～849.9）pg/ml。22例（84.62％）患者出现了脏器肿大（肝肿大23.08％、淋巴结肿大57.69％、脾肿大69.23％），其中5例（19.23％）完成淋巴结穿刺活检，2例（7.69％）确诊Castleman病。17例（65.38％）患者存在皮肤色素沉着，2例（7.69％）皮肤干燥粗糙，2例（7.69％）出现皮肤血管瘤，9例（34.62％）性功能异常，3例（11.54％）糖耐量异常。24例（92.31％）出现内分泌改变，累及内分泌轴数量≤2个的患者有5例（19.23％），数量>2个的患者有19例（73.08％）。

血清免疫电泳M蛋白阳性率84.62％（22/26），19例为λ型（IgA型10例，IgG型6例，同时表达IgA和IgG型3例），2例为κ型（IgG和IgA型各1例），1例为IgA重链型。26例（100％）行骨髓涂片，20例（76.92％）同时行穿刺活检，骨髓涂片浆细胞占比<2％占76.92％（20/26），>2％占23.08％（6/26），骨髓活检中7例（35％）幼稚浆细胞占比>5％。肢体麻木组患者按个体化预后模型分类，低危1例（3.85％），中危13例（50％），高危12例（46.15％）。

3. 治疗与转归：血管外容量负荷增大组中15例患者接受治疗（2例未在我院治疗），2例（13.33％）单独应用糖皮质激素治疗，3例（20％）应用硼替佐米联合糖皮质激素治疗，6例（40％）应用来那度胺联合糖皮质激素治疗，2例（13.33％）应用环磷酰胺联合糖皮质激素治疗，1例（6.67％）应用吗替麦考酚酯联合糖皮质激素治疗，1例（6.67％）应用沙利度胺联合糖皮质激素治疗。其中1例（6.67％）患者化疗后采用auto-HSCT，患者目前恢复良好，状态平稳。上述患者经规律的周期性化疗后，腹胀、水肿或胸闷等不适完全消失，但多发性神经病变及皮肤症状改善不明显，疗效欠佳。截至2023年9月，存活患者13例（86.67％，2例死亡患者考虑高龄自然死亡的可能性大），疗效达到血液学完全缓解（CR_H_）12例（80％）、血液学部分缓解（PR_H_）1例（6.67％）、神经病变改善（R_N_）5例（33.33％）。

肢体麻木组治疗的23例患者（3例未在我院治疗），15例（65.22％）应用来那度胺联合糖皮质激素治疗，4例（17.39％）应用硼替佐米联合糖皮质激素治疗，2例（8.70％）应用吗替麦考酚酯联合糖皮质激素治疗，2例（8.70％）应用美法仑联合糖皮质激素治疗。其中2例（8.70％）患者化疗后采用auto-HSCT，目前达到CR_H_和R_N_。截至2023年9月，患者全部存活，疗效达到CR_H_ 13例（56.52％）、PR_H_ 10例（43.48％）、R_N_ 15例（65.22％）。

## 讨论

POEMS综合征是一种罕见的浆细胞恶液质，它的特点是多发性神经病、单克隆免疫球蛋白、血管外容量负荷增大、内分泌病、脏器肿大和皮肤改变等。虽然根据现有的诊断标准，确诊相对容易[6]，但在发病早期，很多表现往往被误解为伴随症状而被忽视，这种不典型的症状使得早期诊断面临挑战。国内研究数据显示，其误诊率高达85.9％[8]–[9]。在多数情况下，直到患者临床症状均出现或重要脏器受损严重时，医师才会考虑POEMS综合征的可能。这种延迟诊断不仅影响了患者的治疗预后，还会降低生活质量。

有研究认为，浆细胞分泌的VEGF是该病的特异性血清标志物，VEGF靶向内皮细胞，造成细胞肿胀变性，血管通透性增加，大量液体从血管渗出，累积在组织间隙，从而导致组织水肿、积液等特征性表现[10]–[11]。考虑到这一特点，本研究收集并分析了本中心两组患者的数据：一组以血管外容量负荷增大（表现为腹胀、双下肢水肿、颜面水肿等）为首发症状，另一组以临床常见的肢体麻木为首发症状。通过比较分析，重点描述前者的临床特征。结果显示，血管外容量负荷增加组发病年龄较大，就诊时涉及的科室广而杂，超过60％的患者起病至非血液科首诊。此外，从起病到确诊的时间跨度显著，9例患者确诊时间超过12个月，甚至有患者经历长达60个月的诊疗过程，涉及多个科室。这与肢体麻木组形成鲜明对比，后者的确诊过程相对较短。以上数据揭示患者临床早期诊断的困难。在临床表现方面，血管外容量负荷增大组的极个别患者就诊时无周围神经病变。由于体内异常浆细胞负荷低且分布局限、临床检验技术不敏感等原因，该组患者的血清免疫固定电泳阳性率为64.71％。血管外容量负荷增大组似乎更容易满足主要和次要诊断标准，并表现出多系统功能受损，脏器肿大（尤以淋巴结最多见）、水肿、内分泌代谢异常、皮肤改变、视乳头水肿等发生率均高于肢体麻木组。因此在临床诊断中遇到类似患者时，应考虑到更罕见的变异型POEMS综合征[4]–[6]。相比于肢体麻木组，血管外容量负荷增加组的风险分层主要集中在低至中危组别，而高危组患者不足30％。以上提示患者在确诊时可能面临更高的致残率和更低的生活质量。

追溯本研究血管外容量负荷增大组患者的完整诊疗过程，总结误诊的原因如下：①症状特异性差，如心衰、肾功能不全、肝功能不全等都可能导致全身水肿，在临床实践中难以被充分鉴别；②予消肿对症治疗后患者症状得到短暂缓解，造成的假象误导首诊医师不再深入探索潜在的病因；③POEMS综合征早期阶段患者体内单克隆浆细胞负荷低，而且蛋白电泳、血清VEGF检测并非临床常规检查，增加早期确诊的难度。

因此，当处理“不明原因水肿”这一复杂症状，特别是当常规治疗效果不佳时，医师不仅要考虑心源性、肾源性和肝源性等常见病因。同时，也需警惕罕见病的潜在影响。胸腹水的常规检查、生化检查将在诊断过程中起到关键的辅助作用。

总体来说，当代医学的治疗手段已使POEMS综合征患者的6年无进展生存率超过50％[5]，中位生存期达到184个月[12]。尽管如此，这些患者仍面临较高的致残率和较低的生活质量。目前暂无规范的POEMS综合征治疗指南，治疗策略的选择应考虑患者的骨病变和骨髓受累情况，可能涉及放射治疗或全身系统性治疗[13]–[15]，常用的治疗方案有药物（如糖皮质激素、烷化剂、蛋白酶体抑制剂、免疫调节剂、抗VEGF抗体）和auto-HSCT[16]。同时对症支持治疗也是至关重要的：肢体麻木者予营养神经治疗；水肿严重者予利尿、限制水、钠摄入等处理；低蛋白血症者加强营养摄入、考虑输注人血白蛋白等。

本研究38例患者确诊后立即接受了系统性治疗，本中心采取的治疗方案包括单纯的糖皮质激素、基于烷化剂或蛋白酶体抑制的化疗、免疫调节剂及auto-HSCT。经治疗，血管外容量负荷增加组的患者水肿症状完全消失，病情得到缓解。在后续随访期间，未发现任何患者疾病复发，整体治疗效果与肢体麻木组相似。这表明POEMS综合征患者临床症状的异质性对预后影响有限，这与国内相关研究[17]的发现一致。auto-HSCT在本中心治疗POEMS综合征中显示出有效性，其效果与单纯药物治疗相仿。未来的临床研究需要进一步探索auto-HSCT对于不同首发症状或不同风险分层患者的预后影响。值得注意的是，尽管其他症状有所改善，但患者的周围神经损害似乎并未明显好转，其疗效远低于肢体麻木组。这是否与水肿的机制或症状出现的顺序有关，值得进一步研究。综上所述，POEMS综合征的整体预后是乐观的，临床上采取的各种治疗方案均能取得满意的结局。及时、准确的诊断结合系统治疗在延缓疾病进展、提高生活质量方面起到关键作用。

通过本研究，可以看到POEMS综合征起病复杂，涉及科室范围广泛，被误诊和漏诊非常常见[9]，特别是以“血管外容量负荷增大”为首诊症状的患者。在临床实践中，医师对疾病的判断常有滞后，这无疑给改善疾病管理带来了挑战。为了更有效地对抗疾病，本研究认为加强诊疗思维、采用更先进的检测手段和鼓励多学科之间的紧密合作是临床工作的当务之急，以期有效降低误诊率并缩短确诊时间。
